# A redetermination from the original data of the crystal structure of 2-amino-4,6-di­meth­oxy­pyrimidin-1-ium 4-amino­benzoate

**DOI:** 10.1107/S2056989016004321

**Published:** 2016-03-18

**Authors:** Jan Fábry

**Affiliations:** a Institute of Physics of the Czech Academy of Sciences, Na Slovance 2, 182 21 Praha 8, Czech Republic

**Keywords:** crystal structure, redetermination, hydrogen bonding, symmetric hydrogen bonds, refinement constraints, refinement restraints

## Abstract

The title structure, C_6_H_9.5_N_3_O_2_^0.5+^·C_7_H_6.5_NO_2_^0.5−^, which might be named schematically as 2-amino-4,6-di­meth­oxy­pyrimidine-(μ_2_-hydrogen)-4-amino­benzoate to indicate a bridging H atom, has been redetermined from the data published by Thanigaimani, Mu­thiah & Lynch [*Acta Cryst.* (2006), E**62**, o2976–o2978]. The improvement of the present redetermination consists in a released geometry of the primary amine groups, which were originally assumed to be planar, as well as in a redetermination of the position of the hy­droxy H atom. This H atom, whose parameters were originally constrained, turns out to be situated about the centre of the O⋯N hydrogen bond in two disordered positions with occupancies of 0.5 each.

## Abstract 2

The title structure, 2-amino-4,6-di­meth­oxy­pyrimidine-(μ_2_-hydrogen)-4-amino­benzoate, has been redetermined from the data published by Thanigaimani, Mu­thiah & Lynch [*Acta Cryst.* (2006), E**62**, o2976–o2978]. The improvement of the present redetermination consists in a released geometry of the primary amine groups, which were originally been assumed as planar, as well as in a redetermination of the position of the hy­droxy H atom. This H atom, whose parameters were originally constrained, turns out to be situated about the centre of the O⋯N hydrogen bond in two disordered positions, each with 0.5 occupancy.

## Chemical context   

**Figure 1:**
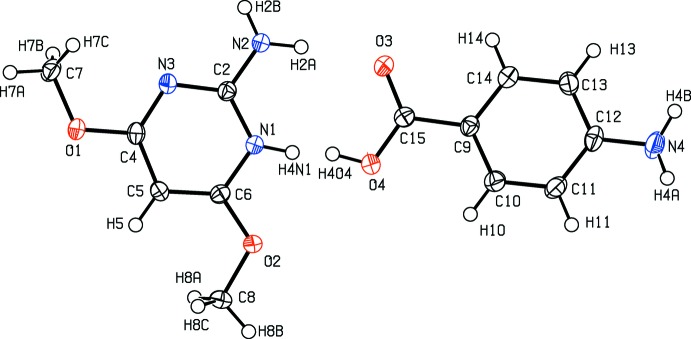
View of the constituent mol­ecules of the title structure after the improved refinement. The displacement ellipsoids are depicted at the 50% probability level. The occupancies of atoms H4N1 and H4O4 are each equal to 0.5.

Structures which contain hydroxyl, secondary and primary amine groups are often determined incorrectly because of an assumed geometry of these groups from which the applied constraints or restraints were inferred. In such cases, the correct geometry is missed as it is not verified by inspection of the difference electron-density maps. Thus, a considerable number of structures could have been determined more correctly – *cf.* Figs. 1 and 2 in Fábry *et al.* (2014 ▸). The inclusion of such structures causes bias in crystallographic databases such as the Cambridge Structural Database (CSD; Groom & Allen, 2014 ▸).
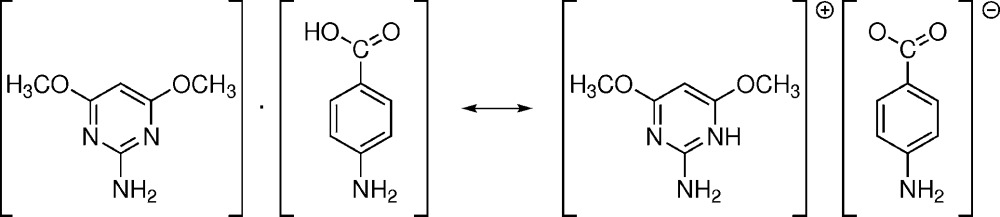


In the course of recalculation of suspect structures which were retrieved from the CSD, defects in the structure determination of 2-amino-4,6-di­meth­oxy­pyrimidine–4-amino­benzoic acid (1/1) by Thanigaimani *et al.* (2006 ▸) have been found; the pertinent CSD refcode is *IFACUO*.

The defects in the original structure concern positional parameters both of the hydroxyl and the primary amine hydrogen atoms, which follow from unsubstanti­ated constraints of these hydrogen atoms. This means that the amine groups were assumed to be planar while the disorder regarding atom H4 was neglected because atom H4 was forced to be situated at atom O4.

The aim of the present article is to demonstrate how the original structure determination can be improved.

## Structural commentary   

The structure of the title compound has been described by Thanigaimani *et al.* (2006 ▸) as 2-amino-4,6-di­meth­oxy­pyrimidine/4-amino­benzoic acid (1/1). In that article, the amine groups (centred on atoms N2 and N4) were assumed to be planar and were refined with distance constraints of N—H = 0.86 Å. For the hydroxyl group O4—H4, atom H4 was refined with a distance constraint of O4—H4 = 0.82 Å [*U*_iso_(H_primary amine_) = 1.2*U*_eq_(N_primary amine_) while *U*_iso_(H4) = 1.5*U*_eq_(O4)].

**Figure 2:**
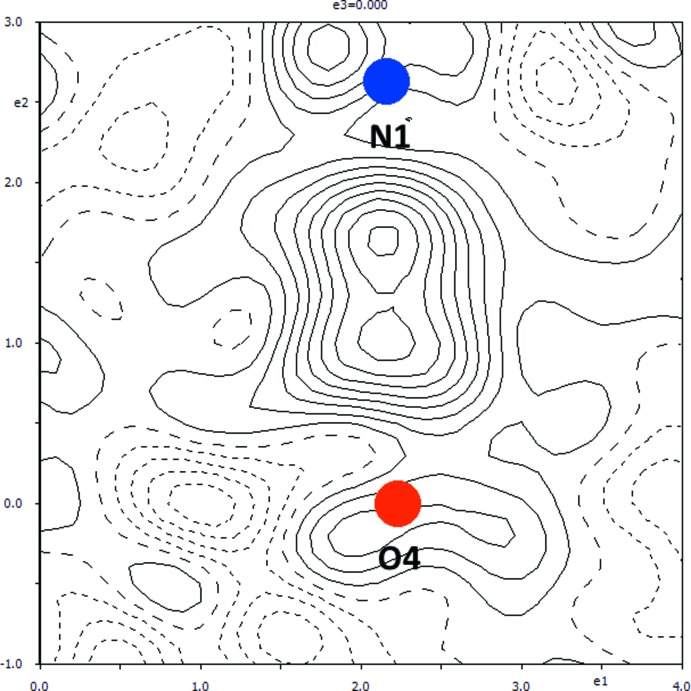
A section of the difference electron-density map for the present redetermined title structure, which shows the build-up of the electron density between the atom O4 (red) and N1 (blue). Positive and negative electron densities are indicated by continuous and dashed lines, respectively. The increment of electron density between neighbouring contours is 0.05 e Å^−3^ (*JANA*2006; Petříček *et al.*, 2014 ▸).

The improved refinement by *JANA*2006 (Petříček *et al.*, 2014 ▸) yielded a non-planar geometry of the primary amine groups and a considerably different position for the hydrogen atom H4. This atom turns out to be disordered over two positions at about the centre of the hydrogen bond O4⋯N1 (Fig. 1 ▸). Thus, the title structure can be envisaged as an example of a structure with a symmetric hydrogen bond where the bridging hydrogen atom is disordered over two positions (see: for example Olovsson *et al.*, 2002 ▸). One of these positions is closer to atom N1 while the other is closer to atom O4, and correspondingly they were labelled as H4*n*1 and H4*o*4. Each of the occupancies of H4*n*1 and H4*o*4 turned out to be equal to 0.5 within the inter­val given by the refined standard uncertainties; *cf.* the section of the electron density map in Fig. 2 ▸. The p*K*a of the conjugate acid to 2-amino-4,6-di­meth­oxy­pyrimidine is equal to 3.36 (Baldwin & van den Broek, 1975 ▸), while p*K*a_1_ and p*K*a_2_ of 4-amino­benzoic acid are equal to 2.50 and 4.87, respectively (*CRC* Handbook of Chemistry and Physics, 2009 ▸). p*K*a_1_ refers to the deprotonation of the hydrogen carboxyl­ate into the carboxyl­ate group, while p*K*a_2_ refers to the deprotonation of the ammonium group into the primary amine group in the solution (*cf.* p*K*a for benzoic acid and aniline are equal to 4.20 and 4.87, respectively; *CRC* Handbook of Chemistry and Physics, 2009 ▸). Thus, 2-amino-4,6-di­meth­oxy­pyrimidine is a weaker acid while 4-amino­benzoic acid is a weaker base. These values favour the formation of the salt rather than of the co-crystal. Since differences in the dissociation constants are relatively mild, the hydrogen atom is situated about the centre of the hydrogen bond N1⋯O4 and the structure in the solid state can be envisaged as a mixture of a co-crystal 2-amino-4,6-di­meth­oxy­pyrimidine–4-amino­benzoic acid (1:1) with a salt 2-amino-4,6-di­meth­oxy­pyrimidin-1-ium 4-amino­benzoate in a 1:1 proportion. Alternatively – as has been stated above – it can be assumed to be a structure with a disordered bridging hydrogen involved in a symmetric hydrogen bond (Olovsson *et al.*, 2002 ▸).

In the recalculated structure determination, the deviation from planarity of the primary amine groups (including the C atoms to which they are attached) is larger for the one that is centred on N4 [C12–N4–H4*a* 115.4 (9), C12–N4–H4*a* 114.5 (9), H4*a*–N4–H4*b* 119.5 (13)°] than on N2 [C2–N2–H2*a* 119.5 (8), C2–N2–H2*b* 119.7 (8), H2*a*–N2–H2*b* 120.7 (12)°]. This is in agreement with the longer bond length for C12—N4 [1.3786 (17) Å] compared to C2—N2 [1.3253 (16) Å].

In a broader sense, the present redetermination emphasizes the importance of careful examination of the difference electron-density maps during structure determinations.

## Supra­molecular features   

**Figure 3:**
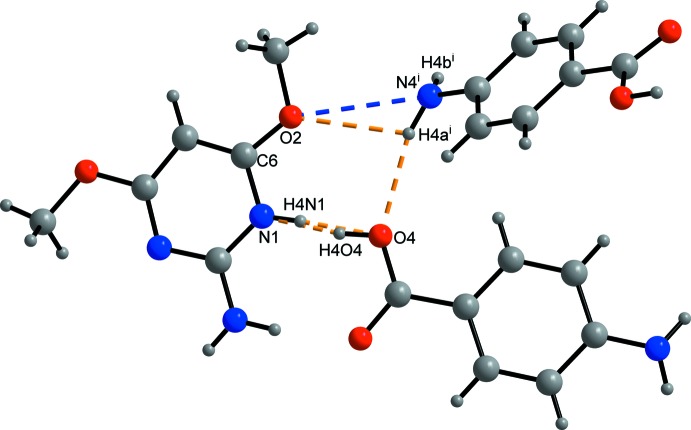
The section of the title structure which shows the graph-set motifs *R*(7)^3^_2_ O4—H4O4⋯N1—C6—O2⋯H4*a* ^i^⋯N4^i^ and O4⋯H4N1—N1—C6—O2⋯H4*a* ^i^⋯N4^i^ [symmetry code: (i) −*x* + 1, *y* − 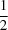, −*z* + 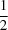; colour code for atoms: grey – C and H, blue – N; red – O; colour code for bonds: black: covalent bonds, dashed orange: H⋯hydrogen-bond acceptor; blue O2—N4: inclusion into the graph-set motif *R*(7)^3^_2_].

The details of the hydrogen bonding and the N—H⋯π-electron ring inter­action involving N4—H4*b* are given in Table 1 ▸. The graph-set motifs (Etter *et al.*, 1990 ▸) were described by Thanigaimani *et al.* (2006 ▸) for the title structure. The graph-set motif 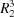
(7) (Fig. 3 ▸) is shown in Fig. 2 ▸ of the article by Thanigaimani *et al.* (2006 ▸) and described there as 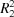
(6).

In the present article, the graph-set motif 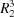
(7) includes the atoms O4—H4*o*4⋯N1—C6—O2⋯H4*a* ^i^—N4^i^ or O4⋯H4*n*1—N1—C6—O2⋯H4*a* ^i^—N4^i^ on a local scale [Fig. 3 ▸; symmetry code (i): −*x* + 1, *y* − 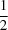, −*z* + 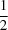].

## Database survey   

The structure determination by Thanigaimani *et al.* (2006 ▸) has been included in the Cambridge Structural Database (Groom & Allen, 2014 ▸) under the refcode *IFACUO*.

## Synthesis and crystallization   

The preparation of the title compound has been described by Thanigaimani *et al.* (2006 ▸).

## Refinement   

Crystal data, data collection and structure refinement details are summarized in Table 2 ▸. All the hydrogen atoms were discernible in the difference electron-density map. The aryl hydrogen atoms were constrained by the constraints C_ar­yl_—H_ar­yl_ = 0.93 Å and *U*_iso_(H_ar­yl_) = 1.2*U*_eq_(C_ar­yl_) while the methyl hydrogens were constrained by the constraints C_meth­yl_—H_meth­yl_ = 0.96 Å and *U*_iso_(H_meth­yl_) = 1.5*U*_eq_(C_meth­yl_). The hydrogen atoms of the primary amine group N2 were constrained by *U*_iso_(H_N2_) = 1.2*U*_eq_(N2). The displacement parameters of the hydroxyl hydrogen H4O4 and of the secondary amine H4N1 were constrained by *U*_iso_(H4O4) = 1.5*U*_eq_(O4) and *U*_iso_(H4N1) = 1.5*U*_eq_(N1) while their positional parameters were refined freely.

The model with the refinement of the occupational factors of H4N1 and H4O4 under the condition that the sum of these occupational factors should equal to 1 resulted in the values 0.499 (25) and 0.501 (25), respectively. Therefore the occupational parameters were set to 0.5 in the final model and not further refined.

## Supplementary Material

Crystal structure: contains datablock(s) global, I. DOI: 10.1107/S2056989016004321/su5289sup1.cif

Structure factors: contains datablock(s) I. DOI: 10.1107/S2056989016004321/su5289Isup2.hkl

Supporting information file. DOI: 10.1107/S2056989016004321/su5289Isup3.smi

CCDC reference: 1465363

Additional supporting information:  crystallographic information; 3D view; checkCIF report

## Figures and Tables

**Table 1:** Hydrogen-bond geometry (Å, °) *Cg*1 is the centroid of the C9–C14 ring.

*D*—H⋯*A*	*D*—H	H⋯*A*	*D*⋯*A*	*D*—H⋯*A*
N1—H4N1⋯O4	0.86 (3)	1.78 (3)	2.6459 (14)	176 (2)
O4—H4O4⋯N1	0.89 (3)	1.77 (3)	2.6459 (14)	170 (3)
N2—H2A⋯O3	0.92 (1)	1.91 (1)	2.8163 (14)	172 (1)
N2—H2B⋯O3^i^	0.88 (1)	2.04 (1)	2.8544 (14)	154 (1)
N4—H4A⋯O4^ii^	0.92 (2)	2.27 (2)	3.1625 (15)	164 (1)
C7—H7B⋯O2^iii^	0.96	2.59	3.4571 (15)	150
N4—H4*b*⋯*Cg*1^iv^	0.89 (2)	2.724 (15)	3.5472 (14)	154.7 (12)

Symmetry codes: (i) 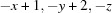; (ii) 
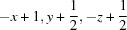
; (iii) 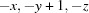; (iv) 
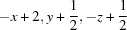
.

**Table 2:** Experimental details

Crystal data	
Chemical formula	C_6_H_9.5_N_3_O_2_^0.5+^·C_7_H_6.5_NO_2_^0.5−^
*M*_r_	292.30
Crystal system, space group	Monoclinic, *P*2_1_/*c*
Temperature (K)	293
*a*, *b*, *c* (Å)	6.6358 (4), 7.5560 (5), 27.4226 (16)
β (°)	94.418 (2)
*V* (Å^3^)	1370.89 (15)
*Z*	4
Radiation type	Mo *K*α
μ (mm^−1^)	0.11
Crystal size (mm)	0.44 × 0.32 × 0.08

Data collection	
Diffractometer	Bruker–Nonius KappaCCD area-detector
No. of measured, independent and observed [*I* > 3σ(*I*)] reflections	14577, 3130, 2302
*R*_int_	0.032
(sin θ/λ)_max_ (Å^−1^)	0.651

Refinement	
*R*[*F* ^2^ > 3σ(*F* ^2^)], *wR*(*F* ^2^), *S*	0.037, 0.084, 1.91
No. of reflections	3130
No. of parameters	208
H-atom treatment	H atoms treated by a mixture of independent and constrained refinement
Δρ_max_, Δρ_min_ (e Å^−3^)	0.21, −0.23

Computer programs: *DENZO* (Otwinowski & Minor, 1997 ▸), *COLLECT* (Hooft, 1998 ▸), *SHELXS97* (Sheldrick, 2008 ▸), *PLATON* (Spek, 2009 ▸), *DIAMOND* (Brandenburg & Putz, 2005 ▸) and *JANA*2006 (Petříček *et al.*, 2014 ▸).

## supplementary crystallographic information

### Crystal data

**Table:**

C_6_H_9.5_N_3_O_2_0.5+·C_7_H_6.5_NO_2_0.5−−	*F*(000) = 616
*M**_r_* = 292.30	*D*_x_ = 1.416 Mg m^−^^3^
Monoclinic, *P*2_1_/*c*	Mo *K*α radiation, λ = 0.71073 Å
Hall symbol: -P 2ybc	Cell parameters from 50 reflections
*a* = 6.6358 (4) Å	θ = 3.5–27.5°
*b* = 7.5560 (5) Å	µ = 0.11 mm^−^^1^
*c* = 27.4226 (16) Å	*T* = 293 K
β = 94.418 (2)°	Block, colourless
*V* = 1370.89 (15) Å^3^	0.44 × 0.32 × 0.08 mm
*Z* = 4	

### Data collection

**Table:**

Bruker–Nonius KappaCCD area-detector diffractometer	2302 reflections with *I* > 3σ(*I*)
Radiation source: Bruker–Nonius FR591 rotating anode	*R*_int_ = 0.032
Graphite monochromator	θ_max_ = 27.5°, θ_min_ = 3.5°
φ and ω scans	*h* = −8→8
14577 measured reflections	*k* = −9→9
3130 independent reflections	*l* = −35→35

### Refinement

**Table:**

Refinement on *F*^2^	50 constraints
*R*[*F*^2^ > 2σ(*F*^2^)] = 0.037	H atoms treated by a mixture of independent and constrained refinement
*wR*(*F*^2^) = 0.084	Weighting scheme based on measured s.u.'s *w* = 1/(σ^2^(*I*) + 0.0004*I*^2^)
*S* = 1.91	(Δ/σ)_max_ = 0.021
3130 reflections	Δρ_max_ = 0.21 e Å^−^^3^
208 parameters	Δρ_min_ = −0.23 e Å^−^^3^
0 restraints	

### Special details

**Table:**

Refinement. This part differs from the original article by Thanigaimani *et al.* (2006). It also differs from the refinement by Thanigaimani *et al.* (2006) by a different threshold for the consideration of the observed diffractions: *F*^2^ > 3σ(*F*^2^) has been used as criterion for observed diffractions by *JANA*2006 which was used for the calculation of the corrected structural model.

### Fractional atomic coordinates and isotropic or equivalent isotropic displacement parameters (Å^2^)

**Table:**

	*x*	*y*	*z*	*U*_iso_*/*U*_eq_	Occ. (<1)
O1	−0.31928 (12)	0.65273 (11)	−0.06555 (3)	0.0245 (3)	
O2	−0.10295 (12)	0.62305 (10)	0.10293 (3)	0.0220 (3)	
N1	0.08195 (15)	0.75282 (13)	0.04894 (4)	0.0190 (3)	
N2	0.29136 (17)	0.87608 (15)	−0.00426 (4)	0.0257 (4)	
N3	−0.01303 (15)	0.76827 (13)	−0.03671 (3)	0.0195 (3)	
C2	0.11662 (18)	0.79867 (15)	0.00265 (4)	0.0187 (4)	
C4	−0.18261 (18)	0.68662 (15)	−0.02786 (4)	0.0194 (4)	
C5	−0.23220 (18)	0.62937 (15)	0.01815 (4)	0.0201 (4)	
C6	−0.09140 (18)	0.66647 (15)	0.05581 (4)	0.0186 (4)	
C7	−0.26609 (18)	0.70054 (18)	−0.11374 (4)	0.0261 (4)	
C8	−0.26910 (18)	0.51184 (16)	0.11446 (4)	0.0244 (4)	
O3	0.54244 (13)	0.93439 (11)	0.08178 (3)	0.0263 (3)	
O4	0.31630 (13)	0.82158 (12)	0.12916 (3)	0.0258 (3)	
N4	0.9485 (2)	1.16699 (16)	0.28973 (4)	0.0366 (4)	
C9	0.60450 (18)	0.96745 (15)	0.16726 (4)	0.0198 (4)	
C10	0.5334 (2)	0.95948 (16)	0.21374 (5)	0.0252 (4)	
C11	0.6456 (2)	1.02612 (16)	0.25407 (5)	0.0286 (4)	
C12	0.8340 (2)	1.10412 (16)	0.24933 (4)	0.0251 (4)	
C13	0.90630 (19)	1.11079 (16)	0.20281 (5)	0.0254 (4)	
C14	0.79433 (19)	1.04458 (15)	0.16262 (5)	0.0227 (4)	
C15	0.48369 (18)	0.90491 (15)	0.12288 (4)	0.0202 (4)	
H2a	0.3827 (19)	0.8954 (17)	0.0219 (5)	0.0308*	
H2b	0.3152 (19)	0.9139 (17)	−0.0336 (5)	0.0308*	
H5	−0.352188	0.570355	0.022827	0.0241*	
H7a	−0.38336	0.691873	−0.13641	0.0391*	
H7b	−0.163259	0.621794	−0.123627	0.0391*	
H7c	−0.216204	0.819832	−0.113329	0.0391*	
H8a	−0.266284	0.403993	0.096019	0.0365*	
H8b	−0.257451	0.484961	0.148775	0.0365*	
H8c	−0.394276	0.572471	0.106262	0.0365*	
H4a	0.878 (2)	1.1906 (19)	0.3166 (6)	0.0439*	
H4b	1.050 (2)	1.237 (2)	0.2829 (5)	0.0439*	
H10	0.408167	0.908373	0.217631	0.0302*	
H11	0.595299	1.019027	0.284732	0.0344*	
H13	1.032145	1.160827	0.198977	0.0305*	
H14	0.845086	1.051013	0.131987	0.0273*	
H4N1	0.157 (5)	0.771 (4)	0.0756 (12)	0.0285*	0.5
H4O4	0.251 (5)	0.797 (4)	0.1004 (12)	0.0387*	0.5

### Atomic displacement parameters (Å^2^)

**Table:**

	*U*^11^	*U*^22^	*U*^33^	*U*^12^	*U*^13^	*U*^23^
O1	0.0216 (5)	0.0332 (5)	0.0179 (5)	−0.0044 (4)	−0.0040 (4)	0.0017 (4)
O2	0.0211 (5)	0.0274 (5)	0.0173 (5)	−0.0059 (4)	0.0009 (4)	0.0032 (4)
N1	0.0188 (6)	0.0218 (6)	0.0161 (5)	−0.0025 (5)	0.0004 (4)	−0.0001 (4)
N2	0.0232 (6)	0.0371 (7)	0.0165 (6)	−0.0107 (5)	−0.0004 (5)	0.0015 (5)
N3	0.0190 (6)	0.0212 (5)	0.0179 (5)	−0.0008 (4)	−0.0012 (4)	−0.0006 (4)
C2	0.0203 (7)	0.0184 (6)	0.0173 (6)	−0.0003 (5)	0.0008 (5)	−0.0010 (5)
C4	0.0188 (7)	0.0188 (6)	0.0201 (6)	0.0027 (5)	−0.0026 (5)	−0.0015 (5)
C5	0.0178 (7)	0.0212 (6)	0.0211 (7)	−0.0029 (5)	0.0000 (5)	0.0002 (5)
C6	0.0212 (7)	0.0171 (6)	0.0175 (6)	0.0015 (5)	0.0024 (5)	0.0007 (5)
C7	0.0260 (7)	0.0347 (8)	0.0169 (7)	0.0014 (6)	−0.0024 (6)	0.0022 (5)
C8	0.0228 (7)	0.0268 (7)	0.0239 (7)	−0.0055 (6)	0.0043 (6)	0.0043 (5)
O3	0.0258 (5)	0.0358 (5)	0.0172 (5)	−0.0057 (4)	0.0016 (4)	−0.0019 (4)
O4	0.0243 (5)	0.0324 (5)	0.0204 (5)	−0.0089 (4)	−0.0002 (4)	0.0003 (4)
N4	0.0480 (8)	0.0393 (7)	0.0209 (6)	−0.0112 (6)	−0.0072 (6)	−0.0028 (5)
C9	0.0236 (7)	0.0177 (6)	0.0176 (6)	0.0004 (5)	−0.0009 (5)	0.0006 (5)
C10	0.0294 (8)	0.0246 (7)	0.0214 (7)	−0.0043 (6)	0.0017 (6)	0.0008 (5)
C11	0.0400 (9)	0.0287 (7)	0.0171 (7)	−0.0048 (6)	0.0017 (6)	0.0005 (5)
C12	0.0341 (8)	0.0189 (6)	0.0207 (7)	0.0012 (6)	−0.0076 (6)	−0.0002 (5)
C13	0.0239 (7)	0.0261 (7)	0.0255 (7)	−0.0033 (6)	−0.0031 (6)	0.0001 (5)
C14	0.0252 (7)	0.0240 (7)	0.0189 (7)	0.0016 (6)	0.0004 (6)	0.0002 (5)
C15	0.0211 (7)	0.0196 (6)	0.0199 (7)	0.0019 (5)	0.0017 (5)	0.0010 (5)

### Geometric parameters (Å, º)

**Table:**

O1—C4	1.3462 (14)	O4—H4O4	0.89 (3)
O1—C7	1.4400 (14)	N4—C12	1.3786 (17)
O2—C6	1.3411 (14)	N4—H4a	0.918 (15)
O2—C8	1.4405 (15)	N4—H4b	0.886 (15)
N1—C2	1.3525 (16)	C9—C10	1.3940 (17)
N1—C6	1.3483 (16)	C9—C14	1.4026 (17)
N1—H4N1	0.86 (3)	C9—C15	1.4821 (16)
N2—C2	1.3253 (16)	C10—C11	1.3799 (17)
N2—H2a	0.914 (13)	C10—H10	0.93
N2—H2b	0.880 (14)	C11—C12	1.3973 (19)
N3—C2	1.3472 (15)	C11—H11	0.93
N3—C4	1.3221 (15)	C12—C13	1.3980 (18)
C4—C5	1.3969 (17)	C13—C14	1.3751 (17)
C5—C6	1.3672 (16)	C13—H13	0.93
C5—H5	0.93	C14—H14	0.93
C7—H7a	0.96	H2a—H2b	1.559 (19)
C7—H7b	0.96	H7a—H7b	1.5677
C7—H7c	0.96	H7a—H7c	1.5677
C8—H8a	0.96	H7b—H7c	1.5677
C8—H8b	0.96	H8a—H8b	1.5677
C8—H8c	0.96	H8a—H8c	1.5677
O3—C15	1.2409 (15)	H8b—H8c	1.5677
O4—C15	1.2998 (15)	H4a—H4b	1.56 (2)
			
C4—O1—C7	117.31 (9)	H8a—C8—H8b	109.47
C6—O2—C8	117.11 (8)	H8a—C8—H8c	109.47
C2—N1—C6	117.63 (10)	H8b—C8—H8c	109.47
C2—N1—H4N1	129 (2)	C15—O4—H4O4	110 (2)
C6—N1—H4N1	114 (2)	C12—N4—H4a	115.4 (9)
C2—N2—H2a	119.5 (8)	C12—N4—H4b	114.5 (9)
C2—N2—H2b	119.7 (8)	H4a—N4—H4b	119.5 (13)
H2a—N2—H2b	120.7 (12)	C10—C9—C14	118.07 (11)
C2—N3—C4	115.62 (10)	C10—C9—C15	122.49 (11)
N1—C2—N2	117.46 (11)	C14—C9—C15	119.39 (11)
N1—C2—N3	124.33 (11)	C9—C10—C11	121.15 (12)
N2—C2—N3	118.20 (11)	C9—C10—H10	119.43
O1—C4—N3	118.71 (10)	C11—C10—H10	119.43
O1—C4—C5	116.29 (10)	C10—C11—C12	120.73 (12)
N3—C4—C5	125.00 (10)	C10—C11—H11	119.64
C4—C5—C6	115.08 (11)	C12—C11—H11	119.64
C4—C5—H5	122.46	N4—C12—C11	120.82 (12)
C6—C5—H5	122.46	N4—C12—C13	120.96 (12)
O2—C6—N1	111.45 (10)	C11—C12—C13	118.18 (11)
O2—C6—C5	126.24 (11)	C12—C13—C14	121.08 (12)
N1—C6—C5	122.31 (11)	C12—C13—H13	119.46
O1—C7—H7a	109.47	C14—C13—H13	119.46
O1—C7—H7b	109.47	C9—C14—C13	120.80 (12)
O1—C7—H7c	109.47	C9—C14—H14	119.6
H7a—C7—H7b	109.47	C13—C14—H14	119.6
H7a—C7—H7c	109.47	O3—C15—O4	122.60 (11)
H7b—C7—H7c	109.47	O3—C15—C9	120.04 (11)
O2—C8—H8a	109.47	O4—C15—C9	117.35 (11)
O2—C8—H8b	109.47	N1—H4N1—H4O4	171 (5)
O2—C8—H8c	109.47	O4—H4O4—H4N1	165 (5)

### Hydrogen-bond geometry (Å, º)

Cg1 is the centroid of the C9–C14 ring.

**Table:**

*D*—H···*A*	*D*—H	H···*A*	*D*···*A*	*D*—H···*A*
N1—H4*N*1···O4	0.86 (3)	1.78 (3)	2.6459 (14)	176 (2)
O4—H4*O*4···N1	0.89 (3)	1.77 (3)	2.6459 (14)	170 (3)
N2—H2A···O3	0.92 (1)	1.91 (1)	2.8163 (14)	172 (1)
N2—H2B···O3^i^	0.88 (1)	2.04 (1)	2.8544 (14)	154 (1)
N4—H4A···O4^ii^	0.92 (2)	2.27 (2)	3.1625 (15)	164 (1)
C7—H7B···O2^iii^	0.96	2.59	3.4571 (15)	150
N4—H4*b*···*Cg*1^iv^	0.89 (2)	2.724 (15)	3.5472 (14)	154.7 (12)

Symmetry codes: (i) −*x*+1, −*y*+2, −*z*; (ii) −*x*+1, *y*+1/2, −*z*+1/2; (iii) −*x*, −*y*+1, −*z*; (iv) −*x*+2, *y*+1/2, −*z*+1/2.
